# Artifacts and pitfalls in shoulder magnetic resonance imaging*

**DOI:** 10.1590/0100-3984.2013.0006

**Published:** 2015

**Authors:** Gustavo Felix Marcon, Tulio Augusto Alves Macedo

**Affiliations:** 1 Master, Physician Assistant at the Service of Radiology and Imaging Diagnosis, Universidade Federal de Uberlândia (UFU), Uberlândia, MG, Brazil.; 2 PhD, Docent Physician at School of Medicine, Universidade Federal de Uberlândia (UFU), Uberlândia, MG, Brazil.

**Keywords:** Shoulder, Magnetic resonance imaging, Musculoskeletal, Artifacts, Pitfalls

## Abstract

Magnetic resonance imaging has revolutionized the diagnosis of shoulder lesions, in many cases becoming the method of choice. However, anatomical variations, artifacts and the particularity of the method may be a source of pitfalls, especially for less experienced radiologists. In order to avoid false-positive and false-negative results, the authors carried out a compilation of imaging findings that may simulate injury. It is the authors’ intention to provide a useful, consistent and comprehensive reference for both beginner residents and skilled radiologists who work with musculoskeletal magnetic resonance imaging, allowing for them to develop more precise reports and helping them to avoid making mistakes.

## Abstract (pt)

A ressonância magnética revolucionou o diagnóstico de lesões do ombro, tornando-se, em muitos casos, o método de escolha. No entanto, as variações anatômicas, artefatos e particularidade do método podem ser fonte de armadilhas, especialmente para radiologistas menos experientes. Para evitar resultados falso-positivos e falso-negativos, foi realizada uma compilação de achados de imagem que podem simular lesões. Pretendemos ser uma referência útil, consistente e abrangente para os residentes iniciantes e radiologistas qualificados que trabalham com ressonância magnética musculoesquelética, a fim de desenvolver relatórios mais precisos e ajudá-los a evitar erros.

## INTRODUCTION

Magnetic resonance imaging (MRI) has revolutionized the diagnosis of injuries of the musculoskeletal system, becoming the method of choice for its ability to depict soft tissue contrast and exceptional facility to acquire images in multiple planes. Additionally, MRI does not use ionizing radiation, which is a huge advantage as compared with other imaging methods such as radiography and computed tomography.

Normal variant is a term that is commonly seen since the early days of the residency training. It is extremely common the observation of anatomical variations at musculoskeletal MRI studies. This is because MRI has an excellent ability to demonstrate tendons, muscles, cartilage, bones, among other structures.

In spite of its enormous advantage in relation to other methods in the musculoskeletal system evaluation, falsepositive diagnoses may sometimes arise due to imaging pitfalls or confusion with normal anatomical variants. Such falsepositive diagnoses might be harmful to the patient. Additionally, radiologists may also get distressed by the mistaken diagnosis.

Many of such misdiagnoses may be caused by lacking knowledge of anatomy and its many variations and particularities. Also, these pitfalls may happen due to characteristics of the physical process of images acquisition, patient positioning and, obviously, the variation of the anatomy itself. Thus, it is essential for radiologists to have a solid understanding of the anatomical structures that vary in appearance, and differentiate them from diseases that affect the musculoskeletal system.

In the present review, the authors describe the most relevant shoulder artifacts and pitfalls observed in the daily clinical routine, with the objective of providing a useful, consistent and comprehensive reference for both beginner residents and skilled radiologists who work with musculoskeletal MRI, allowing for them to develop more precise reports and helping them to avoid making mistakes.

## TENDONS

### Long head of the biceps brachii

The intra-articular portion of the long head of the biceps predominantly originates from the superior labrum (bicepslabral complex or biceps anchor)^(1)^. The tendon attachment to the superior labrum also changes according to contributions from both its anterior and posterior aspects, described as follows^(2)^: 1) posterior labrum only; 2) predominantly from the posterior labrum with small site of attachment to the anterior labrum; 3) identical origins from the anterior and posterior labrum; 4) predominantly from the anterior labrum with small site of attachment to the posterior labrum. Rarely, portions of the biceps tendon may also attach to the capsule itself; to the rotator cuff; or directly to the supraglenoid tubercle^(3-5)^.

In addition to the location of the biceps attachment to the superior labrum, there is variation in the profile of the biceps-labral complex. A type 1 biceps-labral complex is firmly attached to the superior aspect of the glenoid rim. A type II biceps-labral complex has a small sublabral sulcus which may communicate with a sublabral hole and may simulate a labral tear. A type III biceps-labral complex has a meniscus-shaped labrum with a large sulcus^(6)^.

The long head of the biceps brachii tendon crosses the glenohumeral joint, travelling into the rotator interval, where it is intracapsular despite its classification as extrasynovial. Possible intracapsular long biceps tendon imaging pitfalls include intermediate signal intensity, differently from the usual low-signal intensity of this tendon. It occurs due to the magic angle effect. At MRI, a magic angle artifact refers to increased signal intensity at short echo time (TE) sequences (e.g., T1-weighted or PD spin echo sequences) showing tissues with well-ordered collagen fibers towards one direction (e.g., tendon or articular hyaline cartilage). Such an artifact occurs in cases where the angle between the fibers and the magnetic field corresponds to approximately 54.7°^(7)^.

In addition, since the intra-articular portion of the tendon runs superiorly and posteriorly from the bicipital groove to the biceps-labrum complex, it may appear medially shifted, simulating a medial displacement of the tendon.

**Figure 1:**
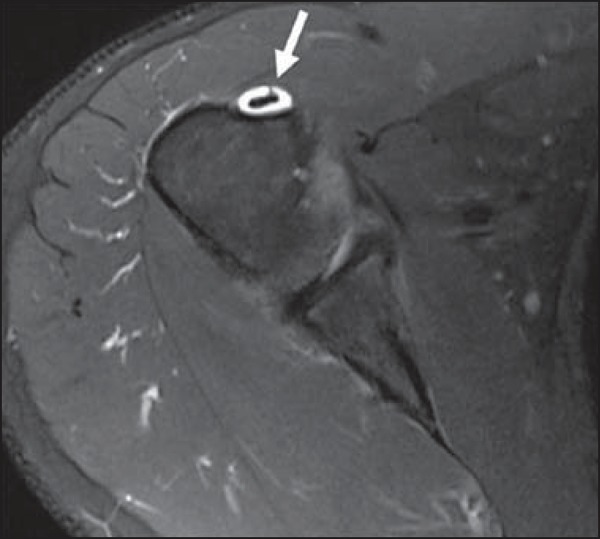
Biceps *vincula* (mesotendon). The biceps tendon obtains its blood supply from a mesotendon (arrow), also called *vincula* tendinum.

**Figure 2:**
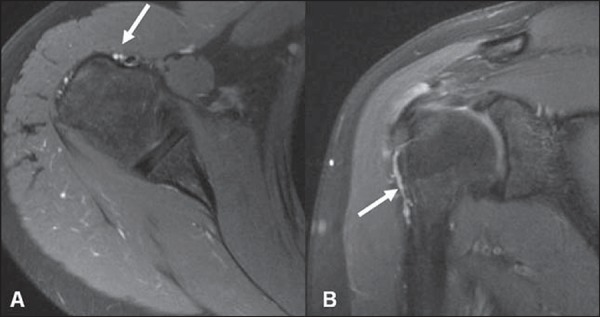
Vessels adjacent to the long head of the biceps tendon (arrows).

The *vincula* of the biceps tendon (synovial bands attaching to the biceps within the tendon sheath) may be seen within the bicipital groove (Figure 1). Blood vessels can also be depicted in the bicipital groove (Figure 2).

**Figure 3:**
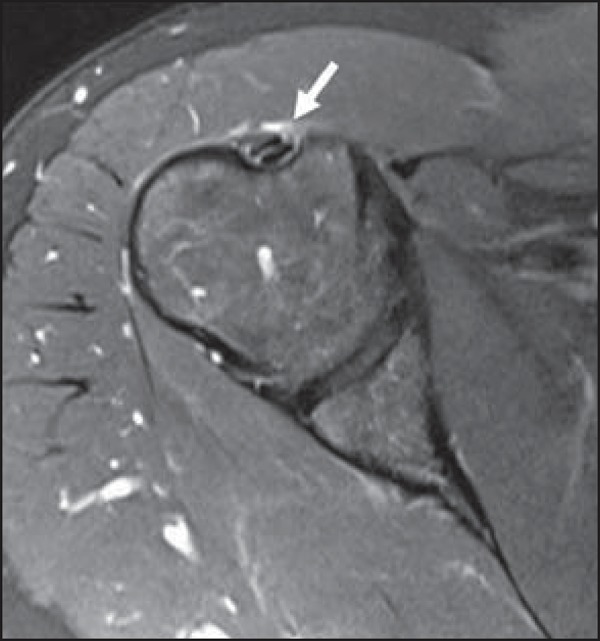
Bifid biceps tendon (arrow).

Some anatomic variations in the origin of the long head of the biceps brachii have been described. The most frequent variation of the biceps brachii is in the number of muscle bellies (Figure 3), although supernumerary heads are frequent, absence of the long or the short head is rarely found^(8)^. Supernumerary heads of the biceps brachii have been described as part of a 3-, 4-, or 5-headed biceps brachii^(9)^. Although bifid or duplicated biceps brachii tendon constitutes a normal variation, it may be mistaken for longitudinal split tendon tears.

### Rotator cuff

The supraspinatus tendon usually inserts into the greater tubercle of the humerus. However, rarely it may be ectopically inserted into the bicipital groove. Mochizuki et al.^(10)^ have demonstrated that the supraspinatus tendon may also insert into the lesser tuberosity of the humerus.

**Figure 4:**
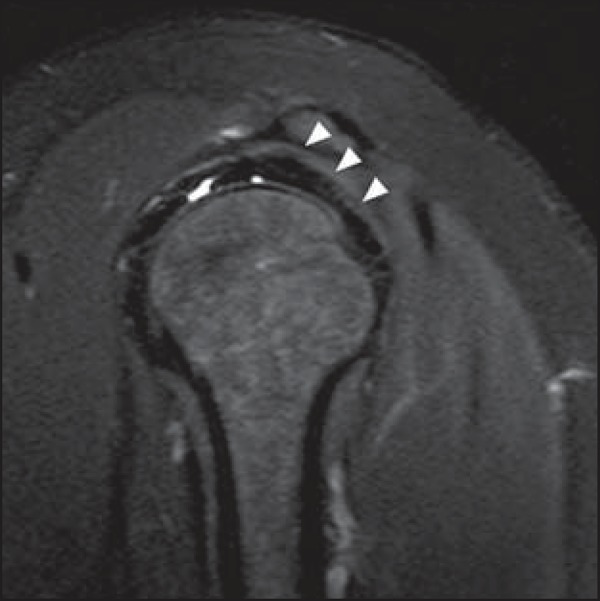
Frequently, in the area of the insertion, one can hardly determine where the supraspinatus tendon ends and where the infraspinatus tendon starts.

Also, Clark et al.^(11)^ have demonstrated the difficulty in separating the fibers of the supraspinatus and infraspinatus tendons (Figure 4). In some cases, one just cannot say exactly which tendon has a tear, but this should not be a source of distress. 

**Figure 5:**
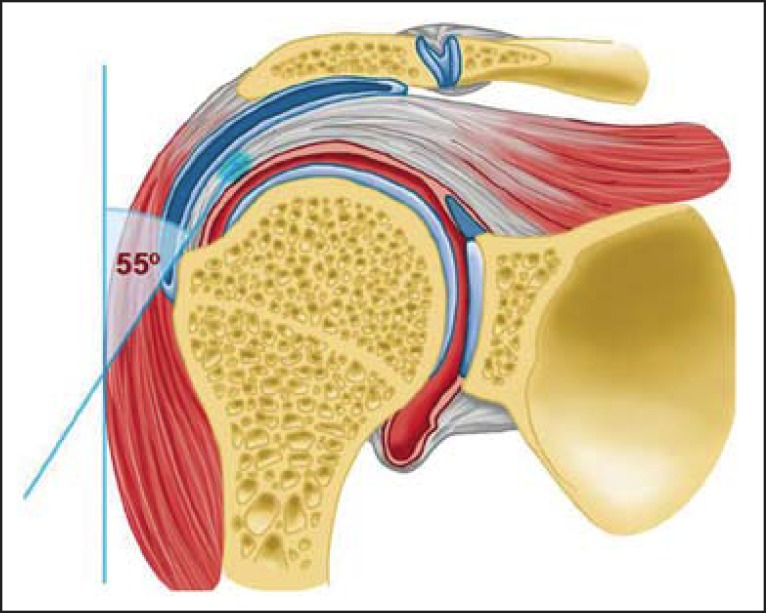
Magic angle and rotator cuff. Increased signal in the supraspinatus tendon at short TE sequence.

**Figure 6:**
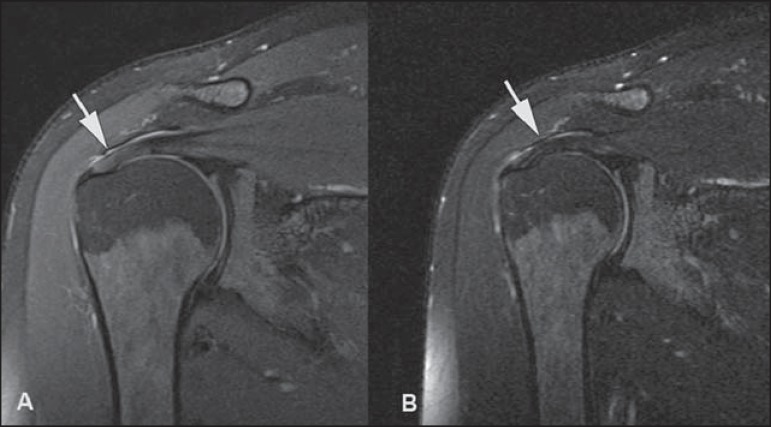
Magic angle and rotator cuff. Coronal PD and T2-weighted images of the supraspinatus insertion. At left, increased signal on the short TE sequence (arrow), which is not seen on the long TE sequence at right (arrow).

A magic angle artifact may also occur in the supraspinatus tendon at short TE spin echo sequences (Figure 5); likewise seen in the intracapsular long head of the biceps brachii. In such cases, T2-weighted sequences are very useful to distinguish between magic angle artifact and rotator cuff tendinosis or partial tendon tear (Figure 6). The area of bright signal within the tendon does not persist or is decreased on T2-weighted images with longer TE^(12)^. 

**Figure 7:**
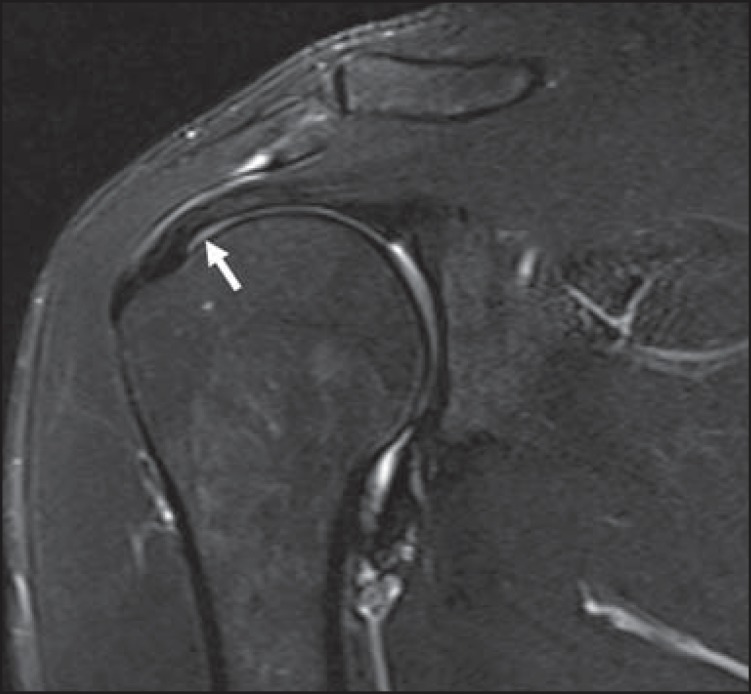
A small sulcus between the osseous insertion of the supraspinatus and the articular cartilage (arrow) is a normal finding.

Another pitfall involving the supraspinatus tendon is the small sulcus of uncovered bone located between the supraspinatus insertion and the joint cartilage (Figure 7). The presence of this sulcus is important because this small area of exposed bone should not be mistaken for a chondral articular surface injury or even a partial tear^(13)^.

## GLENOHUMERAL LIGAMENT AND CAPSULAR VARIANTS

### Inferior glenohumeral ligament

The inferior glenohumeral ligament (IGHL) is the main and the largest stabilizing ligament of the shoulder. It stabilizes the humerus in external and internal rotation during abduction. The IGHL includes two distinct bands: anterior and posterior bands, forming the axillary recess of the joint capsule. The IGHL is usually present as a thickening of the joint capsule and may be mistaken for adhesive capsulitis^(3)^.

### Middle glenohumeral ligament

The middle glenohumeral ligament (MGHL) stabilizes the shoulder during abduction and may present a wide spectrum of anatomic variations. The MGHL may either attach to the anterosuperior labrum, usually just below the origin of the superior glenohumeral ligament, or attach to the anterior scapular neck itself. In this case, it should not be mistaken for capsular injury associated with shoulder instability.

The MGHL may also present a conjoined insertion with the superior glenohumeral ligament (SGHL), the IGHL or even with the long head of the biceps tendon, particularly in cases where the superior glenohumeral ligament is absent.

**Figure 8:**
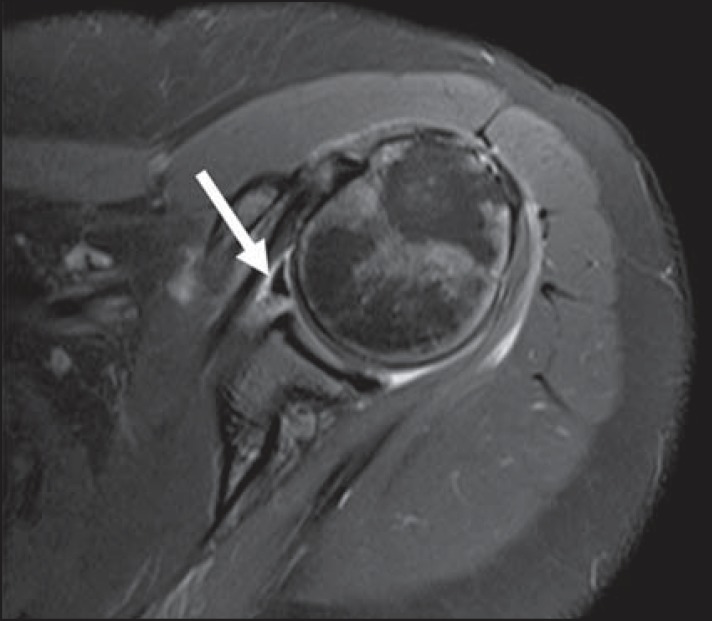
Thickened MGHL (arrow) and absent anterosuperior labrum (Buford complex).

The MGHL may be thickened (a cord-like pattern) or thinned^(14)^. The association of thickened MGHL and absent anterosuperior labrum is called a Buford complex (Figure 8). Compensatory thickening of the MGHL is due to the absent labrum. The frequency of Buford complex approximates to 1.5% of shoulders^(15)^. The Buford complex should not be mistaken for detachment of the anterior superior labrum^(3)^.

Additionally, the MGHL may be duplicated. In such cases, such variation should be distinguished from a SLAP tear^(3)^. The MGHL may be redundant or may join the IGHL or anterior capsule before incorporating onto the base of the lesser tuberosity of the humerus. Similarly to the IGHL, MGHL variations may produce changes in the size and positioning of the capsular recesses.

### Superior glenohumeral ligament

The superior glenohumeral ligament (SGHL) may not be found in 3% of shoulders^(3)^. Usually, the SGHL originates from the anterosuperior labrum, just anterior to the biceps-labral complex, with a conjoined insertion with the MGHL or directly from the proximal biceps tendon^(16)^.

A foramen is typically present between the SGHL and MGHL, which allows the communication between the glenohumeral joint and the subscapularis bursa. In addition, the SGHL is usually thin but may be thickened, particularly in cases where the MGHL is absent.

## SYNOVIAL CAPSULE

The synovial capsule has three variants of the anterior and posterior parts of the glenohumeral joint. The capsule may insert into the glenoid margin (type I), the glenoid neck (type II), or more medially into the scapula (type III)^(17)^. Types II and III should not be mistaken for post-traumatic capsular laxity. Also, there is no correlation between redundancy of the anterior capsule and anterior instability of the shoulder^(3)^.

## LABRAL VARIANTS

Anatomic variations of labral form and contour are commonly found. Usually, such variations occur in the superior half of the labrum, close to the insertion of many of the stabilizing capsular structures, and may easily mistaken for labral or capsular pathology.

**Figure 9:**
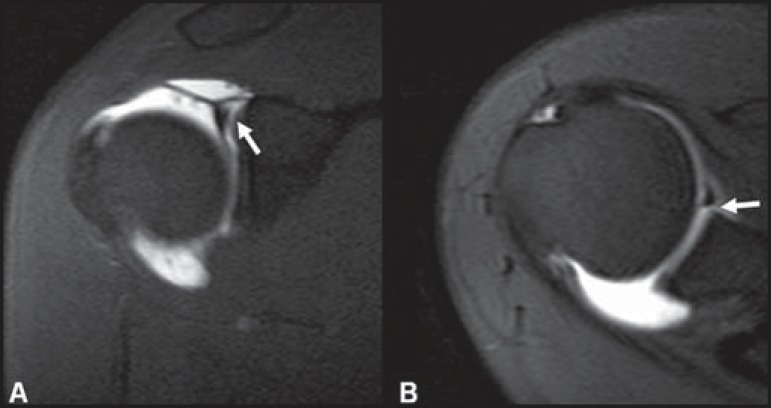
The sublabral foramen (arrows) is an anatomic variation and sometimes it may be not easily differentiated from labral tear.

The sublabral foramen is an anatomic variation located in the anterosuperior labrum, where it is detached from the anterior glenoid margin, allowing for communication between the glenohumeral joint and the subscapular recess (Figure 9). It is important to recognize such a variation at MR arthrography, as it may be misinterpreted as anterior extension of a SLAP tear^(3)^.

**Figure 10:**
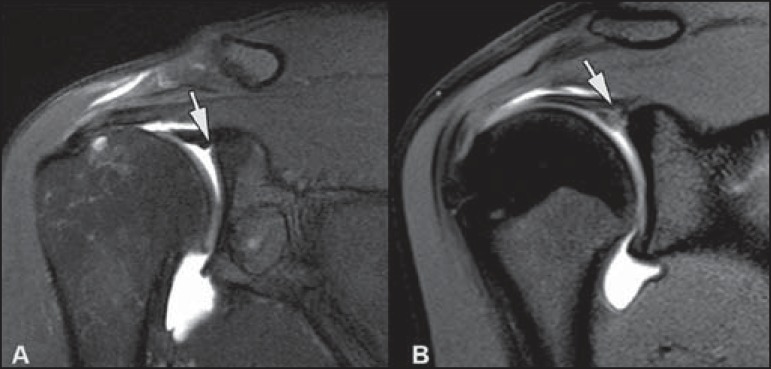
Coronal MR arthrography T1-weighted sequence showing a normal sulcus in the region of the biceps-labral complex (arrow), which is typically parallel to the glenoid (**A**). In SLAP tears, the signal alteration extends towards the substance of the superior labrum (**B**).

The sublabral foramen should be differentiated from the sublabral sulcus. The sublabral sulcus (or recess) is defined as a gap between the biceps-labral complex and the superior glenoid margin. This occurs in type 2 and type 3 biceps-labral complexes where there is a predominant attachment of the biceps medial to the glenoid rim. In this condition, a gap between the biceps-labral complex and the glenoid bone can be seen, particularly at MR arthrography. Therefore, one should be careful so as it is not misdiagnosed as type II SLAP tear. A normal sublabral sulcus should present with similar width and depth. Additionally, the sulcus is not found posterior to the insertion of the LHBT at axial images. At coronal images, the sulcus is parallel to the glenoid rim^(6)^. Signal abnormality spreading from this sulcus is suspicious for SLAP tear (Figure 10).

Finally, intermediate signal intensity may be observed in the region of an expected sulcus at the labrum/glenoid interface. Such intermediate signal intensity is consistent with a histological transition zone between hyaline cartilage and fibrous or fibrocartilaginous tissue, which should also not be mistaken for a labral tear^(3)^.

## JOINT RECESSES AND BURSAE

**Figure 11:**
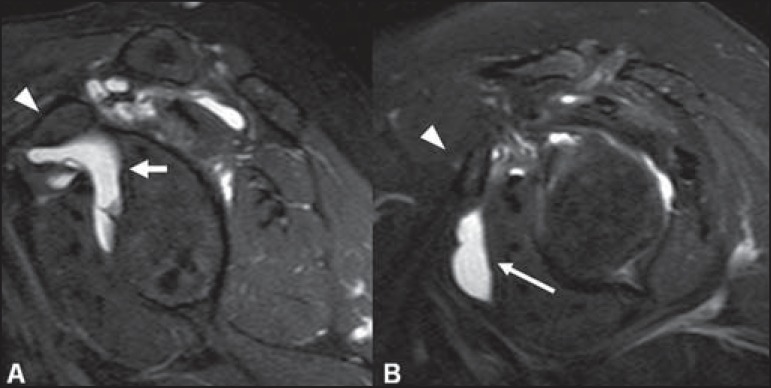
Subscapular recess (**A**) versus subcoracoid bursa (**B**). The subescapular recess communicates with the glenohumeral joint (short arrow). The subcoracoid bursa is located anteriorly to the subscapular tendon and inferiorly to the coracoid process, but does not communicate with the joint cavity. Coracoid process (arrowheads).

The subscapular recess is an extension of the glenohumeral joint located on the posterior and anterosuperior aspects of the subscapularis tendon, typically just below the coracoid process. Therefore, it may be confused for the subcoracoid bursa (Figure 11). The subscapular recess freely communicates with the glenohumeral joint via various possible synovial foramina located between the glenohumeral ligaments. It is should be highlighted that, in healthy shoulders, there is no communication between the glenohumeral joint and the subcoracoid bursa. However, a communication between the subacromial-subdeltoid bursa and subcoracoid bursa might be present^(18)^.

## POSITIONAL VARIATIONS

The supraspinatus and infraspinatus tendons are best imaged with the patient’s shoulder placed in external rotation. Such tendons remain out of the standard at coronal and sagittal imaging as the humerus is internally rotated or externally rotated in excess. In such a condition, the increased tendons overlap may preclude the diagnosis of possible injuries, because the supraspinatus tendon may appear discontinuous.

**Figure 12:**
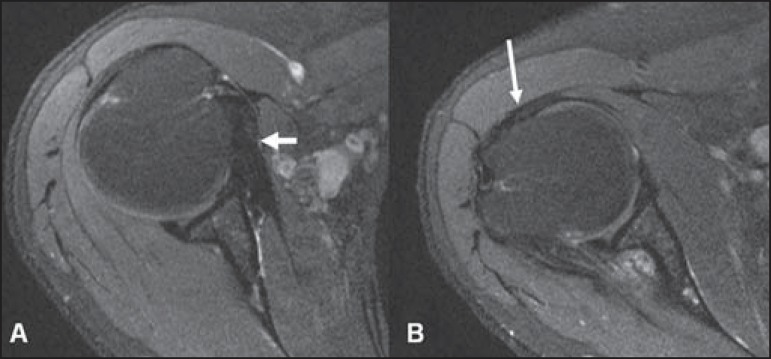
Exaggerated internal rotation on the image **A**, simulating subscapularis tendon thickening (short arrow). Certainly, the tendon is normal (long arrow) with external rotation (**B**).

Also, if there is an exaggerated internal rotation, there may be a false subscapularis tendon thickening or artifactual signal abnormality from the MGHL and capsular structures (Figure 12). Such positioning may also result in abnormal signal intensity of the supraspinatus and infraspinatus, probably due to a magic angle effect.

Internal rotation may also make the assessment of the infraspinatus tendon more difficult, considering that the interposition of muscle and collagen fibers are more appropriately observed with the shoulder in external rotation. This is because of the rotator cuff tendons position parallel to the section plane at coronal oblique images^(19)^.

## ACROMION

**Figure 13:**
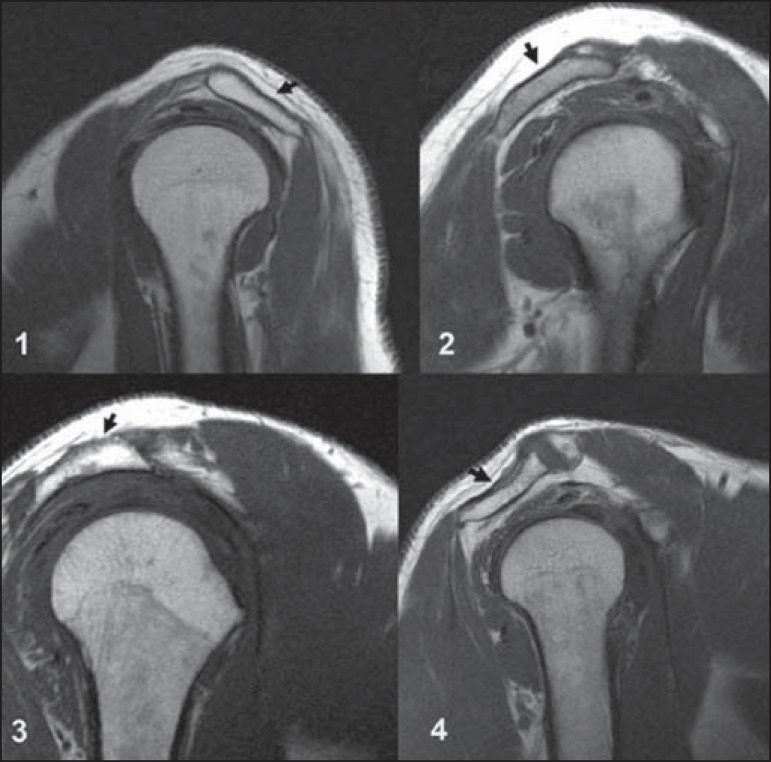
The figures represent each acromion type.

Based on shape, the acromion process has been classified into four types^(20)^, namely, type 1 (straight or flat); type 2 (curved); type 3 (hooked); type 4 (inverted). This fourth type has lately been added and represents a convex or upward pointing undersurface. These morphological acromial variations (Figure 13) may be viewed by using either scapular Y-view radiographs or sagittal oblique and coronal oblique MRI sequences^(21)^.

It is believed that a type 3 acromion or prominent enthesophyte can play a primary role in subacromial impinge-ment syndrome^(20)^ and in injury to the anterior leading edge of the supraspinatus. Other acromial shapes, such as a downward projecting keel spur from the acromion or lateral downsloping of the acromion might also be associated with development of rotator cuff tear^(22)^.

**Figure 14:**
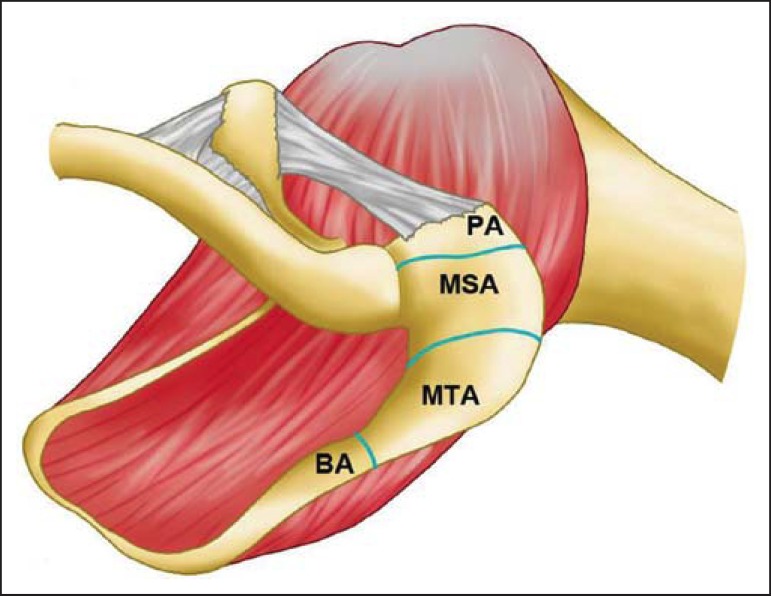
Acromial ossification centers. *Basiacromion* (BA), *meta-acromion* (MTA), *mesoacromion* (MSA) and *preacromion* (PA).

Embryologically, the acromion may be divided into multiple ossification centers, namely, *basiacromion, metaacromion, mesoacromion, and preacromion* (Figure 14). Such ossification centers may be physiologically found during adolescence. After the age of 25, the failure in fusion of these ossification centers may result in the development of an accessory ossicle, the so called os acromiale^(3)^ that is best recognized at axial MRI, where a signal gap is observed between the fat-containing marrow of the distal acromion and the nonfused bone.

**Figure 15:**
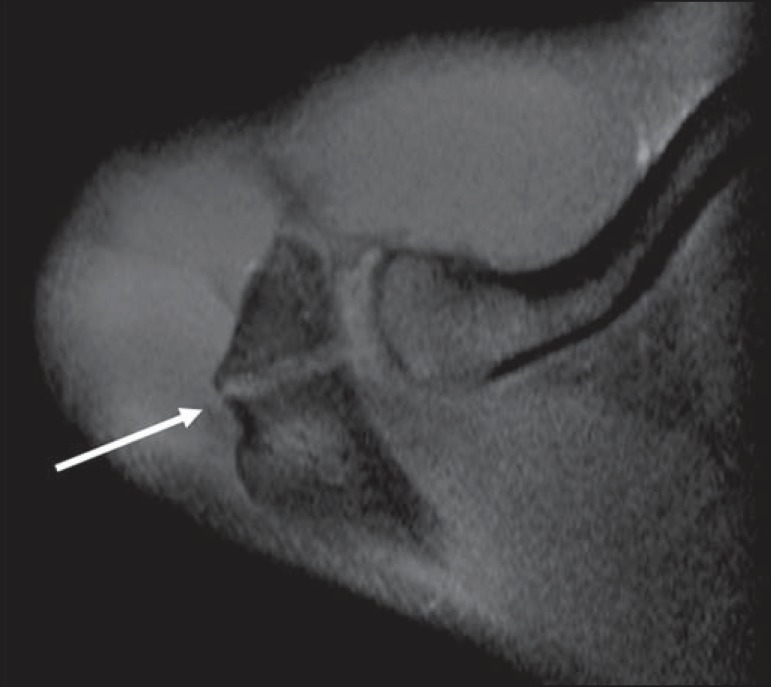
*Os acromiale*.

The *os acromiale* forms a pseudoarticulation with the base of the acromion through the fibrous tissue, periosteum, cartilage or synovium. A second “acromioclavicular” joint may be seen on images (Figure 15), where the *os acromiale* is seen articulating with the clavicle. It is important to identify an *os acromiale* because it plays a role in the development of shoulder impingement symptoms due to inferior displacement at deltoid contraction. Degenerative changes in this pseudoarticulation may occur across the synchondrosis or in association with acromioclavicular degeneration^(3)^.

## HUMERUS

**Figure 16:**
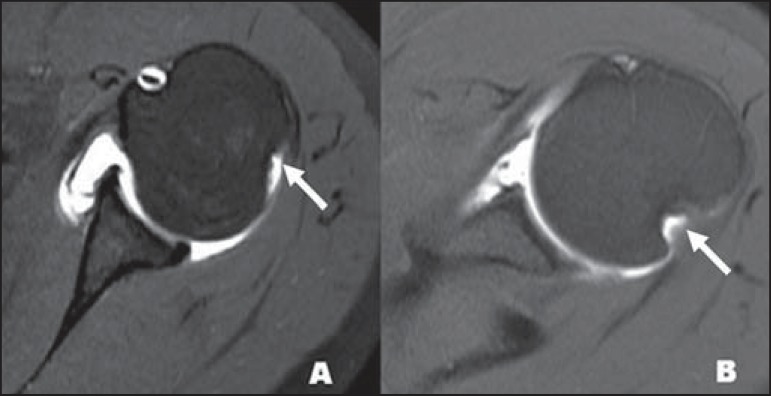
Normal groove in the posterior aspect of the humeral head (**A**). Hill- Sachs defects are visible on the uppermost axial sections (**B**).

A normal groove is frequently found at the posterior aspect of the humerus near the junction of the head and proximal diaphysis, representing a potential pitfall at axial MR imaging, and should not be mistaken for a Hill-Sachs lesion (Figure 16). Such a distinction becomes important in patients with a history of glenohumeral instability, considering that the presence of a Hill-Sachs lesion might warrant surgical treatment. Usually, Hill-Sachs defects are visible on the uppermost axial images of the humerus, superolaterally at 5 mm from the top of the humeral head. The normal anatomic groove of the humerus usually lies at 20 to 30 mm from the superior humeral head and is more medial and posteriorly positioned on axial images. Depth and width of the defect are not considered to be reliable indicators for its differentiation^(23)^. 

## GLENOID

**Figure 17:**
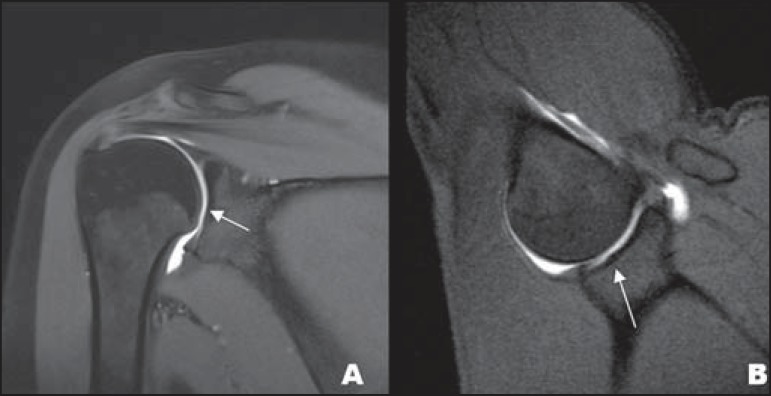
Tubercle of Ossaki. Area of focal subchondral bone thickening with thinned overlying cartilage (arrows).

The shallow glenoid cavity provides increased range of motion of the glenohumeral joint at the cost of stability. This stability is achieved by the surrounding structures, particularly the glenohumeral ligaments, labrum, and rotator cuff. Some imaging abnormalities and variations of the glenoid may be present. At the central region of the glenoid, there is an area of thickened subchondral bone (tubercle of Ossaki) with thinned overlying cartilage (Figure 17). It should not be mistaken for an area of chondral damage or loss^(3)^. On the other hand, in this equivalent area, a smooth focal fullthickness cartilage defect without thickening of the underlying bone may be seen as a normal variant and should not be confused with chondromalacia^(3)^.

The glenoid may present with different shapes on sagittal images, namely, round, ovoid, teardrop-shaped, or pearshaped. The shape relies on variations in the appearance of the glenoid notch, that may be either prominent, diminutive, or absent. The glenoid notch lies on the anterior margin and upper third of the glenoid, usually determining a pear-shaped appearance at sagittal images.

An oval glenoid cavity on sagittal images is produced by the absence of a notch. The labrum is not attached to the bony margin of the glenoid in the region of the notch, resulting in the sublabral recess^(24)^.

In many cases, the glenoid cavity is more concave inferiorly than superiorly^(25)^. Also, the posterior rim of the glenoid may also vary in shape and configuration, with three predominant shapes, as follows: 1) pointed (normal); 2) rounded (lazy-J); 3) triangle-shaped osseous deficiency (delta)^(25)^. Lazy-J and delta shapes are associated with atraumatic posterior shoulder instability^(26)^. At MRI, distinguishing between the different shapes of the posterior labrum may be difficult because there is a mixture of low signal intensity between the cortical bone of the glenoid and the glenoid fibrous tissue.

## BONE MARROW

**Figure 18:**
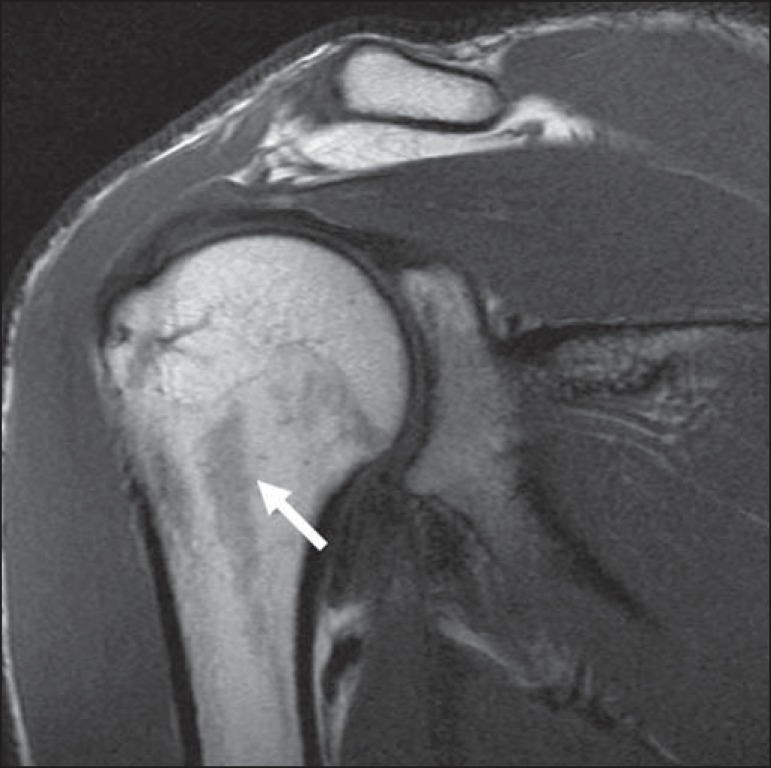
Normal bone marrow striations.

Heterogeneous signal intensity from the bone marrow heterogeneous signal is typically observed in the region of the humeral head and neck. In adult patients, red marrow may be physiologically seen below the physeal scar with slightly hypointense signal at T1- weighted images. Red marrow should not be confused with marrow infiltrative processes, nodules or tumors. In infiltrative processes, the marrow signal becomes markedly low, hypointense to the muscle (Figure 18).

## References

[1] Pal GP, Bhatt RH, Patel VS (1991). Relationship between the tendon of the long head of biceps brachii and
the glenoidal labrum in humans. Anat Rec.

[2] Vangsness CT, Jorgenson SS, Watson T (1994). The origin of the long head of the biceps from the scapula and glenoid
labrum. An anatomical study of 100 shoulders. J Bone Joint Surg Br.

[3] Fitzpatrick D, Walz DM (2010). Shoulder MR imaging normal variants and imaging
artifacts. Magn Reson Imaging Clin N Am.

[4] MacDonald PB (1998). Congenital anomaly of the biceps tendon and anatomy within the
shoulder joint. Arthroscopy.

[5] Yeh L, Pedowitz R, Kwak S (1999). Intracapsular origin of the long head of the biceps
tendon. Skeletal Radiol.

[6] Kwak SM, Brown RR, Resnick D (1998). Anatomy, anatomic variations, and pathology of the 11- to 3-o'clock
position of the glenoid labrum: findings on MR arthrography and anatomic
sections. AJR Am J Roentgenol.

[7] Bydder M, Rahal A, Fullerton GD (2007). The magic angle effect: a source of artifact, determinant of image
contrast, and technique for imaging. J Magn Reson Imaging.

[8] Borghei P, Tehranzadeh J (2010). Bifurcation of the long head of the biceps brachii. Appl Radiol.

[9] Kosugi K, Shibata S, Yamashita H (1992). Supernumerary head of biceps brachii and branching pattern of the
musculocutaneus nerve in Japanese. Surg Radiol Anat.

[10] Mochizuki T, Sugaya H, Uomizu M (2008). Humeral insertion of the supraspinatus and infraspinatus. New
anatomical finding regarding the footprint of the rotador cuff. J Bone Joint Surg Am.

[11] Clark JM, Harryman 2nd DT (1992). Tendons, ligaments, and capsule of the rotator cuff. Gross and
microscopic anatomy. J Bone Joint Surg Am.

[12] Erickson SJ, Cox IH, Hyde JS (1991). Effect of tendon orientation on MR imaging signal intensity: a
manifestation of the "magic angle" phenomenon. Radiology.

[13] Ruotolo C, Fow JE, Nottage WM (2004). The supraspinatus footprint: an anatomic study of the supraspinatus
insertion. Arthroscopy.

[14] Beltran J, Bencardino J, Padron M (2002). The middle glenohumeral ligament: normal anatomy, variants and
pathology. Skeletal Radiol.

[15] Tirman PF, Feller JF, Palmer WE (1996). The Buford complex - a variation of normal shoulder anatomy: MR
arthrographic imaging features. AJR Am J Roentgenol.

[16] Beltran J, Bencardino J, Mellado J (1997). MR arthrography of the shoulder: variants and pitfalls. Radiographics.

[17] Park YH, Lee JY, Moon SH (2000). MR arthrography of the labral capsular ligamentous complex in the
shoulder: imaging variations and pitfalls. AJR Am J Roentgenol.

[18] Schraner AB, Major NM (1999). MR imaging of the subcoracoid bursa. AJR Am J Roentgenol.

[19] Davis SJ, Teresi LM, Bradley WG (1991). Effect of arm rotation on MR imaging of the rotator
cuff. Radiology.

[20] Bigliani LU, Ticker JB, Flatow EL (1991). The relationship of acromial architecture to rotator cuff
disease. Clin Sports Med.

[21] Peh WC, Farmer TH, Totty WG (1995). Acromial arch shape: assessment with MR imaging. Radiology.

[22] Edelson JG, Taitz C (1992). Anatomy of the coraco-acromial arch. Relation to degeneration of the
acromion. J Bone Joint Surg Br.

[23] Richards RD, Sartoris DJ, Pathria MN (1994). Hill-Sachs lesion and normal humeral groove: MR imaging features
allowing their differentiation. Radiology.

[24] Prescher A, Klümpen T (1997). The glenoid notch and its relation to the shape of the glenoid cavity
of the scapula. J Anat.

[25] Mulligan ME, Pontius CS (2005). Posterior-inferior glenoid rim shapes by MR imaging. Surg Radiol Anat.

[26] Weishaupt D, Zanetti M, Nyffeler RW (2000). Posterior glenoid rim deficiency in recurrent (atraumatic) posterior
shoulder instability. Skeletal Radiol.

